# Reduction of rejection‐related emotions by transcranial direct current stimulation over right ventrolateral prefrontal cortex in borderline personality disorder: A double‐blind randomized pilot study

**DOI:** 10.1111/pcn.13792

**Published:** 2025-02-08

**Authors:** Alessandro Lisco, Alessia Gallucci, Chiara Fabietti, Annalisa Fornaroli, Carlo Marchesi, Emanuele Preti, Paolo Riva, Chiara De Panfilis, Leonor Josefina Romero Lauro

**Affiliations:** ^1^ Department of Medicine and Surgery, Unit of Neuroscience University of Parma Parma Italy; ^2^ Ospedale Maria Luigia, Monticelli Terme Parma Italy; ^3^ IRCCS Fondazione Don Carlo Gnocchi Onlus Milan Italy; ^4^ Department of Mental Health Local Health Agency Parma Italy; ^5^ Department of Psychology University of Milan‐Bicocca Milan Italy; ^6^ NeuroMi, University of Milano‐Bicocca Milan Italy

**Keywords:** borderline personality disorder, emotional regulation, prefrontal cortex, social exclusion, transcranial direct current stimulation

## Abstract

**Aims:**

Borderline personality disorder (BPD) patients show negative emotional reactions to both excluding and including social scenarios, with levels normalizing only during extreme inclusion. Prior research among healthy individuals highlights the right ventrolateral prefrontal cortex (rVLPFC) role in regulating emotional responses to social exclusion, since transcranial direct current stimulation (tDCS) of rVLPFC decreases rejection‐related emotions following social exclusion. This pilot study investigated whether, in BPD patients, tDCS over the rVLPFC reduces rejection‐related emotions not only after social exclusion but also after fair social inclusion.

**Methods:**

Forty BPD patients randomly received either real or sham tDCS on rVLPFC before participating in the Cyberball paradigm, which involved phases of inclusion, exclusion, and over‐inclusion. Participants self‐reported their level of rejection‐related emotions following each phase.

**Results:**

Transcranial direct current stimulation reduced rejection‐related emotions during both social exclusion and fair inclusion, but not during over‐inclusion. Specifically, those in the Real tDCS group exhibited comparable emotional responses to fair and over‐including scenarios, unlike those in the Sham group who experienced heightened rejection‐related emotions during fair inclusion compared to over‐inclusion.

**Conclusions:**

Transcranial direct current stimulation over the rVLPFC reduces BPD patients' tendency to feel rejected both in fairly including and excluding scenarios. These findings confirm the rVLPFC involvement in emotional regulation and highlight a therapeutic potential for tDCS in moderating BPD's typical heightened rejection‐related emotional responses to fairly including scenarios. This study supports the application of tDCS in BPD treatment, providing new insights into neuromodulatory interventions that can aid BPD patients to better regulate their emotions during varying social scenarios.

Borderline personality disorder (BPD) is a severe mental health condition affecting approximately 0.7%–2.7% of the general population, with prevalence ranging from 12% among outpatients to 22% among inpatients.^1^, ^2^

Disturbed interpersonal relationships are a core feature of BPD: patients typically establish intense, unstable relationships, with idealization and devaluation of others, unrealistic fear of abandonment,^3^ and difficulties in social adjustment.^4^, ^5^, ^6^ Social rejection exacerbates these difficulties, increasing the risk of maladaptive emotional reactions,^7^ aggressive behaviors,^8^ suicide attempts,^9^ and substance use.^10^

## Misinterpretation of social scenarios in BPD

Increasing evidence suggests that BPD patients feel rejected even in objectively including social scenarios, showing biased views of others as rejecting.^11^, ^12^, ^13^ This hypothesis has been tested using experimental tasks such as Cyberball, a virtual ball‐tossing game that reproduces different levels of social inclusion by manipulating the percentage of tosses participants receive from two other co‐players.^14^, ^15^ Participants can experience fair inclusion (33% of balls received), ostracism/exclusion (receiving the ball only once at the beginning), or over‐inclusion (45% of balls received, more often than would be expected in a fair game where all players receive an equal number of tosses). Extensive Cyberball research demonstrates that, due to humans' fundamental need to belong, social exclusion leads to negative emotional experiences.^16^ BPD patients, in contrast to healthy controls (HC), experience an increased threat to their need to belong not only when excluded but also during fair inclusion.^17^, ^18^, ^19^, ^20^ After both scenarios, they exhibit higher levels of negative affects^21^, ^22^ and greater negative emotions toward others^8^ than non‐BPD controls. Only in conditions of over‐inclusion, where inclusion is more than fair, do BPD patients report levels of rejection‐related emotions comparable to those experienced by HC in typical inclusion scenarios.^22^

Overall, these findings suggest the tendency to misinterpret social scenarios in BPD: BPD individuals feel excluded even in objectively including social interactions.^20^, ^22^, ^23^ This social‐cognitive pattern likely fosters the cascade of interpersonal difficulties associated with the disorder.^6^ Thus, more inquiry is needed into potential strategies that can help BPD patients overcome their feelings of exclusion.

## The regulatory role of the right ventrolateral prefrontal cortex (rVLPFC) following social exclusion

Functional neuroimaging studies identified cerebral networks involved in regulating individuals' emotional and behavioral responses to social exclusion,^24^, ^25^, ^26^ which include, among other brain regions, the ventrolateral prefrontal cortex, especially its right portion (rVLPFC). The rVLPFC plays a major role in down‐regulating negative emotions,^27^ mainly via a ventral neural system usually activated during automatic generation of affective responses.^28^, ^29^ The rVLPFC was found to mediate social pain, anxiety, and affective instability elicited by social exclusion.^30^, ^31^, ^32^, ^33^, ^34^, ^35^, ^36^ The activation of the rVLPFC seems to promote the emotional regulation required to respond adaptively and seek social reconnection.^37^ Consistently, an enhanced rVLPFC activation negatively correlates with the intensity of self‐reported social pain,^38^ while a lower activation characterizes individuals high on rejection sensitivity.^39^ As compared to HC, BPD patients exhibit different activation patterns of the rVLPFC in various emotion‐related tasks. For instance, BPD patients exhibit heightened rVLPFC activation following critical social feedback^40^ and when performing an emotional Stroop task,^41^ suggesting that they require greater activation to engage in emotional regulation efforts and to override interference from emotional information. BPD patients also show decreased rVLPFC activation during a cognitive reappraisal task, indicating a diminished capacity to reinterpret negative emotions.^42^ Although the mechanisms underlying these seemingly contradictory findings need to be elucidated, overall existing research points to a functional alteration of rVLPFC in BPD.

The role of the rVLPFC in regulating rejection‐related emotions and social pain was further demonstrated by applying non‐invasive brain stimulation techniques (NIBS), such as transcranial direct current stimulation (tDCS). In healthy participants, increasing the excitability of the rVLPFC using anodal tDCS decreased social pain and aggressive reactions following the Cyberball social exclusion condition.^43^, ^44^ Conversely, down‐regulating this area using cathodal tDCS increased the levels of negative emotions experienced following social exclusion.^45^ Furthermore, anodal tDCS over the rVLPFC decreased negative emotion ratings when people reappraise emotions elicited by seeing images of social exclusion: such effect, albeit present, is reduced when evaluating negative images unrelated to social exclusion, confirming the specific role of the rVLPFC in regulating emotions induced by social rejection.^34^, ^46^, ^47^, ^48^

An open question is whether this type of stimulation may reduce negative emotions following social exclusion also in BPD patients. Accordingly, tDCS over the rVLPFC could potentially enhance the regulation of rejection‐related emotions in BPD, both in exclusion and inclusion situations. Other NIBS protocols in BPD samples have targeted the dorsolateral and dorsomedial prefrontal cortex, either in single or multiple sessions, and found reductions in other important symptoms of BPD psychopathology, such as impulsivity, aggression, and affective dysregulation.^49^, ^50^, ^51^, ^52^ However, to our knowledge, no study has yet evaluated the emotional responses to anodal tDCS over the rVLPFC in BPD patients following social exclusion, inclusion, and over‐inclusion.

## The present study

The present study assesses whether anodal tDCS over the rVLPFC, compared to sham stimulation, influences rejection‐related emotions in BPD patients under varying Cyberball conditions – social inclusion, exclusion, and over‐inclusion. Based on the evidence that BPD patients often feel rejected even in fairly inclusive situations, but not when overincluded,^20^, ^22^, ^23^ we hypothesize that anodal tDCS over the rVLPFC would mitigate rejection‐related emotions following both social exclusion and fair inclusion, while this effect would be absent during over‐inclusion scenarios. Additionally, based on previous findings that among HC tDCS decreases social pain and aggressive reactions following social exclusion,^43^, ^44^ and that BPD individuals feel threatened in their need to belong even in over‐including scenarios,^22^, ^23^ we explored whether tDCS over the rVLPFC could also alleviate other negative effects of social exclusion in BPD patients, such as threats to fundamental needs, social pain, and aggressive behaviors.^8^, ^53^, ^54^

## Methods

### Participants

Forty BPD patients (36 females, mean age = 33.3 ± 13.2) were recruited among outpatients seeking treatment at an Italian Department of Mental Health. The sample size was based on a priori power analysis (see Supplementary Material [SM]). All participants received a detailed description of the study and gave written informed consent to participate. Personal data were de‐identified and associated with a numeric code, preserving participants' anonymity.

Inclusion criteria were: age 18–65; BPD DSM‐5^55^ diagnosis via Structured Clinical Interview for DSM‐5, Personality Disorders (SCID‐5‐PD^56^). Exclusion criteria were: psychosis, active mood disorder or active substance use, diagnosed by Structured Clinical Interview for DSM‐5 (SCID‐5‐CV^57^); cognitive impairment; tDCS exclusion criteria (unstable medical conditions, neurologic/heart diseases, presence of metallic implants/pacemakers/acoustic prostheses^58^); previous participation in Cyberball studies.

The study protocol was approved by the Area Vasta Emilia Nord Ethical Authority of Emilia‐Romagna region (Italy) (PG0076150_2018), according to Helsinki's Declaration. This experimental psychopathology study does not include any health‐related clinical outcome but just a laboratory outcome. Therefore, it was not registered as a clinical trial.

### Procedure

Before the experiment, participants completed self‐report questionnaires assessing baseline characteristics: Inventory of Personality Organization;^59^ Effortful Control Scale;^60^ Difficulties in Emotion Regulation Scale;^61^ Acceptance and Action Questionnaire;^62^ Justice Sensitivity Questionnaire;^63^ Adult Rejection Sensitivity Questionnaire^64^ (for details and values see SM, Table S1).

Patients were randomized in a 1:1 ratio to receive either sham (20 patients, 18 females; ‘Sham’ group) or active tDCS (20 patients, 18 females; ‘Real’ group). Randomization was conducted based on the order of entry into the study, using a previously generated randomization list.

Then, participants underwent the tDCS protocol. After about 5 min of stimulation, patients participated in the Cyberball paradigm, a virtual ball‐tossing game.^15^ The game lasted around 15 min, so for the group receiving real tDCS, tDCS was administered while performing the task. Participants self‐reported their subjective reactions after each Cyberball condition.

#### tDCS protocol

Real anodal tDCS was delivered through a BrainStim stimulator (EMS, Bologna, Italy) for 20 min, with 1.5 mA as intensity. In the sham condition the stimulation lasted only 20 s, with 30 s of rump‐in/out as in the real condition to maintain the same physical sensations, thus keeping the participants blind to the tDCS condition.^58^, ^65^ An intracefalic montage was used with the anode (25 cm^2^, current density of 0.06 mA/cm^2^) placed over the rVLPFC and the cathode (35 cm^2^, current density of 0.04 mA/cm^2^) placed on the contralateral supraorbital region. We opted for electrodes of different sizes to enhance the focality of stimulation.^58^ The tDCS parameters, including electrodes size, montage, and current density, are in line with recommendations for tDCS use^58^ and clinical applications.^66^, ^67^ Furthermore, we used the same tDCS parameters from our previous studies, where tDCS successfully modulated rejection‐related emotions in healthy participants exposed to the Cyberball paradigm. Blinded investigators, using pre‐programmed codes (numeric labels: 1 and 2), administered real/sham tDCS.

#### Cyberball experiment

Participants believed they were engaging in a mental visualization exercise with two online players (computer‐controlled confederates). All participants experienced, in a fixed order, first inclusion (33% of throws), then exclusion (10% of throws), and lastly over‐inclusion (45% of throws). Post each Cyberball condition, patients rated perceived received throws (0%–100%) and feelings of exclusion (1–7 Likert scale).

After each Cyberball condition, patients self‐reported their subjective reactions through the following questionnaires, tapping primary and secondary exploratory outcomes measures.

##### Primary outcome measure

Rejection‐related emotions were evaluated through the Rejected Emotions Scale (RES^68^), 18 items (1–7 Likert scale) measuring participants' positive (happiness, three items) and negative (anger, sadness, anxiety, feeling rejected, feeling hurt; three items each) emotions. An overall RES index was computed based on the mean of all items after reversing scores of the positive‐formulated items.

##### Exploratory outcome measures

Threats to fundamental needs were measured by a shortened version of the Need‐Threat Scale (NTS^14^), four items (1–7 Likert scale) assessing participants' psychological distress in terms of perceived loss of control, loss of self‐esteem, loss of belongingness, and meaningful existence.

Perceived pain was measured by the Pain Face Scale (PFS^69^), a single pictorial item showing six faces expressing different levels of pain, from ‘no hurt’ to ‘the worst hurt’.

Aggressive temptations were measured by Aggressive Temptation Scale‐short version (ATS^70^), six items (1–7 Likert scale) assessing the degree to which participants felt tempted to engage in aggressive (e.g. humiliate/ignore/insult another person) or prosocial (e.g. smile to another person) behaviors during the Cyberball paradigm.

At the end of the experiment, participants were extensively debriefed.

### Data analysis

Data were analyzed through spss Statistics (Version 27.0; IBM Corp, Armonk, NY, USA). The two groups (Real vs Sham tDCS) were compared on demographics, comorbidity, and medication status using *χ* ^2^ and Student's *t*‐test. Changes in participants' reactions (RES, PFS, NTS, and ATS scores) to the three Cyberball conditions following either real or sham tDCS over the rVLPFC were tested using a repeated‐measures 2×3 mixed model analysis of variance (anova), with tDCS conditions (two levels: Real vs Sham) as the between‐subjects factor and the Cyberball conditions (three levels: inclusion, exclusion, and over‐inclusion) as the within‐subjects factor.

For all the models examined, if the assumption of sphericity was violated (Mauchly's test), based on the epsilon value, either Greenhouse–Geisser's (*ε* < 0.75) or Hyundt‐Felt's (*ε* > 0.75) correction was used. Simple effects analyses with Bonferroni's correction for multiple comparisons were used to evaluate significant main and interaction effects.

## Results

### Sample characteristics

The two groups (Real vs Sham) did not differ in age, gender distribution, comorbidity with other mental disorders, and medication status (Table 1).

**Table 1:** Sample characteristics

Sample features	Real group (*n* = 20)	Sham group (*n* = 20)	*P*
Age (mean [SD])	34.25 (±15.35)	32.45 (±11.05)	0.67^†^
Sex (female) (*n* [%])	18 (90%)	18 (90%)	1^‡^
Occupational status (*n* [%])			
Student	7 (35%)	7 (35%)	0.77^§^
Employed	9 (45%)	9 (45%)	
Unemployed	4 (20%)	3 (15%)	
Retired	0 (0%)	1 (5%)	
Psychiatric comorbidity (lifetime) (*n* [%])			
Personality disorders	3 (15%)	6 (30%)	0.23^‡^
Narcissistic	1 (5%)	3 (15%)	
Histrionic	0 (0%)	1 (5%)	
Dependent	1 (5%)	0 (0%)	
Avoidant	0 (0%)	1 (5%)	
Obsessive‐compulsive	0 (0%)	1 (5%)	
Other mental disorders	11 (55%)	8 (40%)	0.26^‡^
Mood disorders	2 (10%)	3 (15%)	
Eating disorders	6 (30%)	4 (20%)	
Substance use disorders	2 (10%)	1 (5%)	
Obsessive compulsive disorder	1 (5%)	0 (0%)	
Psychopharmacotherapy (*n* [%])¶			
Any pharmacotherapy	18 (90%)	19 (90%)	1^‡^
Antidepressants	12 (60%)	13 (65%)	
Antipsychotics	7 (35%)	9 (45%)	
Mood stabilizers	10 (50%)	14 (70%)	
Anxiolytics	10 (50%)	9 (45%)	

Values are mean (SD) or *n* (%) where specified.

^†^ Student's *t* test for independent samples.

^‡^ Fisher's exact test.

^§^ *χ* ^2^ test.

^¶^ Medication status was assessed prior to the experiment, during the screening process, by asking the participants.

### Cyberball manipulation check

anova showed a significant Cyberball condition effect on perceived throw percentages (*F*[1.32, 50.001] = 105.5, *P* < 0.001, *η* ^2^_p_ = 0.74) and feelings of being ignored/excluded (*F*[2, 76]=160.11, *P* < 0.001, *η* ^2^_p_ = 0.81). Patients reported varying percentages of throws (exclusion: 5.53 ± 2.35; inclusion: 25.25 ± 1.89; over‐inclusion: 62.20 ± 4.34) and levels of feeling ignored/excluded (exclusion: 6.23 ± 0.23; inclusion: 2.7 ± 0.24; over‐inclusion: 1.38 ± 0.16), confirming successful Cyberball manipulation, irrespective of Real or Sham group assignment (Group × Condition interaction, respectively: *P* = 0.476; *P* = 0.100). Furthermore, while there was no significant main effect of the tDCS condition for perceived throw percentages (*P* = 0.797), for levels of feeling ignored/excluded the Real group reported lower feelings than the Sham group (*P* = 0.042).

Supplementary analyses further clarified that tDCS condition had no effect on rejection appraisal, as assessed by perceived throw percentages (see SM, Fig. S1, Tables S2, S3).

#### Primary outcome measure: Rejection‐related emotions

The anova showed a significant main effect of the Cyberball condition on RES total score, with participants reporting progressively lower levels of rejection‐related emotions from the exclusion condition to the inclusion and then over‐inclusion condition (Table 2).

**Table 2:** Effect of tDCS stimulation, Cyberball conditions and their interactions on the levels of rejection‐related emotions, social pain, perceived threats to fundamental needs and aggressive temptations

	Real tDCS (*n* = 20)			Sham tDCS (*n* = 20)					
	Inclusion	Exclusion	Over‐inclusion	Inclusion	Exclusion	Over‐inclusion			
Measures	Mean (SD)	Mean (SD)	Mean (SD)	Mean (SD)	Mean (SD)	Mean (SD)	Cyberball	tDCS	Cyberball × tDCS
RES	2.36 (0.76)	3.7 (1.2)	2.04 (0.67)	3.15 (0.90)	4.65 (1.3)	2.12 (0.82)	*F*[1.856, 70.536] = 69.75 *η* ^2^_p_ = 0.65 ***P*** < **0.001** ^†^	*F*[1, 38] = 7.44 *η* ^2^_p_ = 0.16 ***P*** = **0.010** ^§^	*F*[1.856, 70.536] = 3.29 *η* ^2^_p_ = 0.08 ***P*** = **0.047** ^¶^ ^,^ ^††^
NTS	3.86 (1.23)	5.28 (1.33)	3.05 (0.69)	4.50 (0.88)	6.03 (0.94)	2.78 (1.13)	*F*[1.834, 69.704] = 84.43 *η* ^2^_p_ = 0.69 ***P*** < **0.001** ^†^	*F*[1, 38] = 2.63 *η* ^2^_p_ = 0.06 *P* = 0.113	*F*[1.834, 69.704] = 3.55 *η* ^2^_p_ = 0.09 ***P*** = **0.038** ^‡‡^
Pain Face Scale	1.45 (1.05)	2.00 (1.59)	0.85 (0.87)	1.25 (1.12)	2.85 (1.42)	1.15 (1.14)	*F*[1.871, 671.097] = 21.39 *η* ^2^_p_ = 0.36 ***P*** < **0.001** ^‡^	*F*[1, 38] = 1.24 *η* ^2^_p_ = 0.03 *P* = 0.272	*F*[1.871, 671.097] = 2.67 *η* ^2^_p_ = 0.07 *P* = 0.079
ATS prosocial	4.52 (1.82)	3.25 (1.86)	4.95 (1.79)	4.23 (2.04)	2.55 (2.08)	4.37 (2.37)	*F*[2, 76] = 17.85 *η* ^2^_p_ = 0.32 ***P*** < **0.001** ^‡^	*F*[1, 38] = 1.02 *η* ^2^_p_ = 0.03 *P* = 0.319	*F*[2, 76] = 0.23 *η* ^2^_p_ = 0.01 *P* = 0.794
ATS aggressive	1.63 (1.1)	2.47 (1.59)	1.28 (0.70)	1.58 (0.86)	3.13 (1.85)	1.63 (1.34)	*F*[2, 76] = 21.92 *η* ^2^_p_ = 0.37 ***P*** < **0.001** ^‡^	*F*[1, 38] = 0.99 *η* ^2^_p_ = 0.03 *P* = 0.325	*F*[2, 76] = 1.31 *η* ^2^_p_ = 0.03 *P* = 0.276

*P*‐values of significant effects and interactions are in bold.

Significant post‐hoc (all *P*_s_ < 0.050) expressed with the following apex indices.

^†^ Exclusion > Inclusion > Over‐inclusion.

^‡^ Exclusion > Inclusion = Over‐inclusion.

^§^ Real < Sham.

^¶^ Real: Exclusion > Inclusion = Over‐inclusion; Sham: Exclusion > Inclusion > Over‐inclusion.

^††^ Inclusion and Exclusion: Real < Sham; Over‐inclusion: Real = Sham.

^‡‡^ Exclusion: Real < Sham; Inclusion and Over‐inclusion: Real = Sham.

ATS, Aggressive Temptation Scale; NTS, Need‐Threat Scale; RES, Rejected Emotion Scale; tDCS, transcranial direct current stimulation.

Secondly, the analysis showed a significant main effect of tDCS condition: Patients in the Real group reported lower rejection‐related emotions than those in the Sham group (Table 2).

**Fig. 1:**
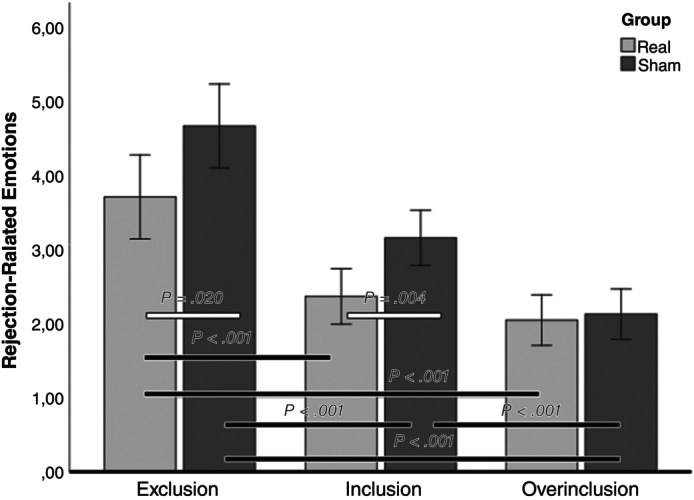
Cyberball × tDCS interaction for rejection‐related emotions (RES scores). This bar graph illustrates the significant differences in rejection‐related emotions across different Cyberball conditions, comparing results between groups (indicated by white bars) and within groups (shown by black bars). Error bars represent the 95% confidence interval (CI). RES, Rejected Emotions Scale; tDCS, transcranial direct current stimulation.

Thirdly, a significant Cyberball × tDCS interaction also emerged (Fig. 1). Patients in the Real group, as compared to Sham group, reported fewer rejection‐related emotions in both the exclusion (*P* = 0.020) and the inclusion (*P* = 0.004) conditions, while the two groups did not differ in the over‐inclusion condition (*P* = 0.736). Thus, tDCS was effective in decreasing rejection‐related emotions not only following social exclusion, but also after fair inclusion. The Group × Condition interaction showed that, while participants in the Sham group experienced significantly fewer rejection‐related emotions in the over‐inclusion condition compared to inclusion (*P* < 0.001), participants in the Real group reported no differences in rejection‐related emotions between the fair inclusion and over‐inclusion conditions (*P* = 0.494). This suggests that in BPD patients, real tDCS effectively reduced the rejection‐related emotions elicited by fair inclusion to levels comparable to those observed in the over‐inclusion condition (Table 2; Fig. 1). These effects were not influenced by rejection appraisal and were confirmed even when controlling for age, occupational status, and comorbidities (see SM, Fig. S2, Tables S4, S5). Supplementary analyses were also performed on medication effects and on single RES subscales (see SM, Tables S6, S7).

#### Exploratory outcome measures

##### Threats to fundamental needs

For NTS scores, anova revealed a main effect only of the Cyberball condition. Post‐hoc comparisons showed that perceived threats to fundamental needs differed significantly across all three Cyberball conditions, with over‐inclusion < inclusion < exclusion (all *P*_s_ < 0.001). There was no main effect of tDCS condition (*P* > 0.050; Table 2).

**Fig. 2:**
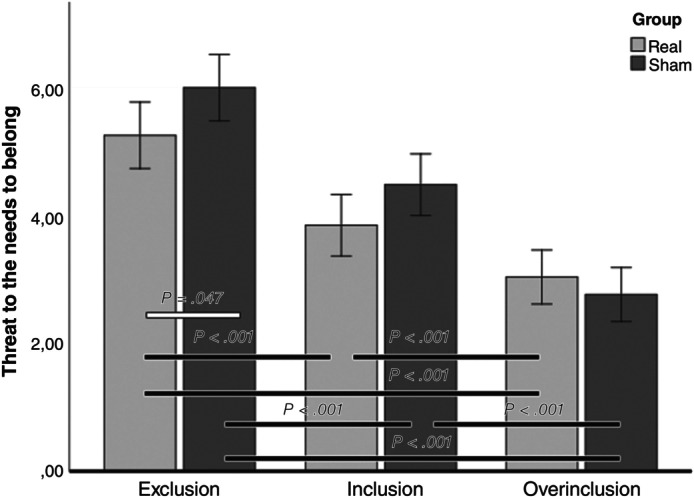
Cyberball × tDCS interaction for perceived Threats to Fundamental Needs (NTS scores). The bars show the presence of a significant difference in the Cyberball conditions between (white bars) or within (black bars) the two groups. Error bars represent 95% confidence interval (CI). NTS, Need‐Threat Scale; tDCS, transcranial direct current stimulation.

However, there was a significant Cyberball × tDCS interaction. Post‐hoc comparisons showed that the two study groups significantly differed in the exclusion condition, where the Real group reported lower NTS scores than the Sham group (*P* = 0.047), but not in the inclusion and over‐inclusion conditions (respectively *P* = 0.067, *P* = 0.359) (Table 2; Fig. 2).

##### Perceived social pain

For PFS scores, anova showed only a main effect of Cyberball condition, with higher pain scores in the social exclusion condition as compared with the inclusion and over‐inclusion (*P*_s_ < 0.001), with no significant differences between the inclusion and the over‐inclusion conditions (*P* = 0.173). No other main or interaction effects were found (*P*_s_ >0.05; Table 2).

##### Aggressive temptations

For ATS scores, Cyberball condition showed a main effect on ATS prosocial and aggressive behaviors. Prosocial scores were lower in social exclusion than inclusion and over‐inclusion (*P*_s_ < 0.001), with no difference between inclusion and over‐inclusion (*P* = 1). Aggressive behaviors were higher in social exclusion than inclusion and over‐inclusion (*P*_s_ < 0.001), with no difference between inclusion and over‐inclusion (*P* = 1). No other significant effects were observed (*P* > 0.050; Table 2). In this BPD sample, tDCS did not reduce aggressive temptations triggered by social exclusion.

## Discussion

To our knowledge, this is the first study showing that, in BPD, stimulating the rVLPFC with anodal tDCS at 1.5 mA effectively downregulated rejection‐related emotions following both social exclusion and inclusion, but not after over‐inclusion.

Previous studies indicated that during the Cyberball paradigm BPD patients, as compared to HC, exhibit a specific ‘difficulty with being socially accepted’,^5^ with higher negative emotions than control subjects not only after exclusion but also after inclusion.^11^, ^13^, ^21^, ^22^, ^23^ Accordingly, in our study, the group receiving sham tDCS exhibited greater rejection‐related emotions during inclusion compared to over‐inclusion. Importantly, patients receiving anodal stimulation over the rVLPFC showed lower levels of rejection‐related emotions compared to sham, not only during episodes of actual exclusion but also following fair inclusion. This suggests that tDCS over the rVLPFC may mitigate the characteristic tendency of BPD individuals to emotionally react to inclusive social interactions as though they are being excluded.

Our findings align with existing literature on the role of the rVLPFC in regulating negative emotions following social exclusion.^43^, ^44^, ^45^, ^46^ Moreover, our study extends available knowledge by showing that the effect of anodal tDCS over the rVLPFC on emotion regulation not only affects healthy subjects but also extends to clinical populations. As an interference technique, tDCS effectively probes the role of the rVLPFC in promoting emotion regulation under all those circumstances subjectively experienced as socially rejecting. This includes instances of actual social exclusion in non‐clinical populations and scenarios of fair social inclusion for groups such as those with BPD, who typically require significantly stronger signals of inclusion to achieve the level of emotional stability that non‐clinical populations experience during standard inclusive interactions.^22^, ^23^ The rVLPFC is part of a broader network responsible for emotion regulation.^28^ Given the low spatial resolution of tDCS, it is possible that our stimulation also affected other brain areas. Therefore, the role of the rVLPFC in regulating rejection‐related emotions isn't exclusive or exhaustive.

The anodal stimulation of the rVLPFC had no effect on the levels of rejection‐related emotions reported by participants in the over‐inclusion condition, likely because this condition does not evoke rejection feelings in BPD patients. This observation reinforces the role of the rVLPFC in modulating emotions triggered by negatively valenced stimuli or contexts, but not in scenarios eliciting positive emotions.^31^, ^48^ Similarly, in non‐clinical populations, real anodal tDCS over rVLPFC doesn't alter rejection‐related emotions following actual social inclusion, likely because healthy individuals typically do not respond negatively to genuine inclusion.^43^, ^44^, ^45^

Importantly, tDCS did not affect objective rejection perception (i.e. percent of balls received during Cyberball). Furthermore, the reduction of rejection‐related emotions by tDCS was not explained by variations in the (cognitive) rejection perception during the experiment. This finding indicates that the stimulation, in this BPD sample, specifically affected the emotional rather than the cognitive‐perceptual domain, further suggesting a distinct pattern of information processing in BPD. While BPD patients showed no differences in the perception of their objective level of inclusion throughout the Cyberball experiment regardless of the tDCS condition, they still experienced heightened rejection‐related emotions at the subjective level in the sham condition. Real anodal tDCS over the rVLPFC reduced this biased emotional response, indicating that the intervention modulated their subjective emotional processing rather than their cognitive appraisal of social inclusion.

As exploratory goals, we assessed the efficacy of rVLPFC tDCS in reducing threats to fundamental human needs, social pain, and aggressive temptations in BPD. Real tDCS over the rVLPFC reduced BPD patients' threats of fundamental needs after social exclusion, reinforcing rVLPFC's role in regulating negative emotions from exclusion.^43^ However, tDCS had no impact on improving satisfaction for fundamental needs after social inclusion, a deficit observed in BPD. Previous studies indicated BPD patients feel threats not just after social exclusion but also in inclusion and over‐inclusion conditions.^20^, ^23^ This enduring threat perception in BPD, irrespective of actual social acceptance, might be a persistent, core feature of BPD psychopathology,^3^ unaffected by a single session of rVLPFC brain stimulation.

In investigating the impact of tDCS over the rVLPFC in BPD, both Real and Sham groups reported increased aggressive temptations and social pain after social exclusion compared to inclusion and over‐inclusion, with no tDCS modulation observed. This aligns with prior evidence of heightened pain and aggression in BPD individuals after social exclusion.^8^, ^23^ However, it contradicts our previous findings in non‐clinical participants, where anodal tDCS on rVLPFC reduced social pain and aggression post‐exclusion.^43^, ^44^ Potential explanations for this absence of an effect include the possibility that a single tDCS session or 1.5 mA of intensity might be insufficient for achieving observable effects in BPD. Future studies with multiple sessions and/or increased intensity could explore this hypothesis. Additionally, approach motivations like behavioral aggression may be associated with greater left frontal activity,^71^, ^72^, ^73^, ^74^ and left VLPFC anodal stimulation has been shown to increase aggression in healthy subjects.^75^ Alternatively, prefrontal areas other than VLPFC, such as DLPFC, could be more involved in modulating cognitive control and impulsivity after ostracism.^31^, ^76^ Previous studies stimulating right DLPFC in BPD patients, indeed, have shown decreased anger, aggression, and impulsivity, suggesting it may promote functional control strategies.^49^, ^51^, ^77^, ^78^, ^79^

This study's results hold relevant clinical applications. First, they extend the effectiveness of tDCS employment also to BPD patients. While psychotherapies for BPD decrease symptoms' severity at a clinically important level, this is not the case for psychosocial dysfunction: improvements in social functioning with different psychotherapeutic approaches are small, do not meet the criterion of minimal clinically relevant change, and are not sustained over time after the end of the intervention.^80^ In particular, the tendency to readily feel excluded by others and negatively emotionally overreact is a core determinant of interpersonal dysfunction in BPD. Thus, the present preliminary results may pave the way for the use of tDCS protocols, combined with concurrent cognitive/emotional training,^81^ as adjuvant treatments to the classical therapies for BPD patients to promote satisfactory interpersonal functioning.

Moreover, these results may extend to other clinical populations with heightened rejection sensitivity, such as those with social anxiety disorder, and highlight the potential of anodal tDCS of the rVLPFC in reducing emotional distress related to normal inclusion across various disorders.

Notably, in this study, the tDCS was delivered during the Cyberball paradigm, based on the principle of state‐dependency of tDCS effects,^82^, ^83^, ^84^ whereby stimulation effects are influenced by the activation state of the targeted brain area. Our results support the value of coupling tDCS with a concurrent task to activate the target network and achieve specific effects.^85^ Although state‐dependency is well known experimentally, clinical applications often do not consider it, and tDCS is frequently administered at rest.^86^ Future studies using experience sampling methods could determine whether multiple sessions of anodal tDCS over the rVLPFC combined with the Cyberball paradigm can improve emotion regulation in real‐life interpersonal interactions in BPD patients (e.g. via a far‐transfer effect).

Secondly, this study underscores the utility of considering the rVLPFC as a target for NIBS in mental disorders. While most NIBS studies have focused on the DLPFC due to its role in executive processes, inhibitory control, and voluntary emotion regulation, recent findings suggest the VLPFC's critical involvement in automatic emotional reactivity. For example, Wolkenstein et al.^85^ applied anodal tDCS over the left DLPFC in a sham‐controlled study on BPD patients and HC during a task involving emotional distraction from negative stimuli. In contrast, the VLPFC is implicated in detecting the emotional valence of stimuli, producing affective states, and automatically regulating emotional responses.^76^

Although our study cannot definitively rule out effects on the dorsal PFC, our findings suggest that targeting the more ventral portion may be particularly effective in addressing heightened emotional reactivity in BPD patients, as seen in their responses to social exclusion and inclusion. Similarly, recent studies on addiction have highlighted the ventral and medial portions of the PFC as promising targets for NIBS protocols aimed at modulating cue‐related reactivity.^87^ These findings suggest that focusing on these regions could be a fruitful avenue for future research on emotional regulation.

Our study has some limitations. First, we included medicated BPD patients. Previous studies found that psychotropic medications may have confounding effects on tDCS outcomes, increasing or decreasing both acute and after‐effects of the stimulation.^88^, ^89^ In our sample, the two study groups did not differ regarding the number of subjects who were or were not taking medications. However, we cannot totally exclude that those medications somehow interfered with the tDCS effects. Another limitation is the low spatial resolution of tDCS that, as discussed before, prevents us from firmly defining the neural correlates of rejection‐related emotion regulation. Over‐inclusion effects are inherently less consistent and robust compared to exclusion.^90^ The fixed order of Cyberball manipulations in our study may have compounded this, with residual effects from the exclusion phase potentially influencing participants' responses during over‐inclusion. This limitation should be addressed in future studies by counterbalancing conditions to disentangle the carryover effects of exclusion on subsequent phases.

In conclusion, our findings indicate that in BDP patients, tDCS applied to the rVLPFC reduces rejection‐related emotions not only in contexts of clear social exclusion but also in normally‐inclusive settings. Issues such as the need for interpersonal closeness and an intolerance to abandonment and rejection are central to BPD psychopathology. These challenges may stem from a specific bias toward overlooking positive social cues that would typically indicate social acceptance.^5^, ^23^, ^91^, ^92^, ^93^, ^94^ If these findings are corroborated, they could endorse the use of tDCS in BPD as a targeted therapeutic strategy to enhance the regulation of rejection‐related emotions triggered by a misinterpretation of social interactions as exclusionary in this severe patient population.

## Disclosure statement

The authors declare no potential financial conflict of interest. E. P. is a member of the executive board and chair of the research committee of the International Society for Transference‐Focused Psychotherapy (ISTFP). C. D. P. is a member of the executive board of the ISTFP and Chair of the applied TFP committee, and board member and president‐elect of the European Society for the Study of Personality Disorder.

## Author contributions

C. D. P., P. R., E. P. and L. J. R. L. designed the study and A. L., C. F. and A. F. collected the data. A. L. and A. G. analyzed the data and all authors interpreted the data. A. L. and A. G. created all figures and tables and wrote the first draft of the manuscript. C. D. P., P. R., E. P., L. J. R. L., C. M. finalized the last version of the manuscript. All authors approved the submitted version.

## Supporting information


**Figure S1.** Interaction between rejection appraisal (perceived ball tosses received) and actual ball tosses received for tDCS groups.
**Figure S2.** Interaction between rejection appraisal (perceived ball tosses received) and Rejection‐related emotions for tDCS groups.
**Table S1.** Sample baseline measures and comparison.
**Table S2.** Regression Model of perceived percentage of ball tosses received for tDCS group.
**Table S3.** Regression Model of perceived percentage of ball tosses received for tDCS group and actual ball tosses received.
**Table S4.** Regression Model of Rejection‐related emotions for tDCS group and rejection appraisal (i.e. perceived percentage of ball tosses received).
**Table S5.** Effect of tDCS stimulation, Cyberball conditions and their interactions on rejection‐related emotion controlled for age, occupational status and presence of psychiatric comorbidities.
**Table S6.** Effect of tDCS stimulation, Cyberball conditions and their interactions on rejection‐related emotion controlled for different pharmacological categories.
**Table S7.** Effect of tDCS stimulation, Cyberball conditions and their interactions on subscale of Rejected Emotion Scale.
