# Supplementary material for: Reduction of rejection‐related emotions by transcranial direct current stimulation over right ventrolateral prefrontal cortex in borderline personality disorder: A double‐blind randomized pilot study
Source: Psychiatry Clin Neurosci. 2025 Feb 8;79(5):239–47. doi: 10.1111/pcn.13792 (PMC12047065; doi:10.1111/pcn.13792)
Supplement: Supplementary file 1 — Figure S1. Interaction between rejection appraisal (perceived ball tosses received) and actual ball tosses received for tDCS groups. Figure S2. Interaction between rejection appraisal (perceived ball tosses received) and Rejection‐related emotions for tDCS groups. Table S1. Sample baseline measures and comparison. Table S2. Regression Model of perceived percentage of ball tosses received for tDCS group. Table S3. Regression Model of perceived percentage of ball tosses received for tDCS group and actual ball tosses received. Table S4. Regression Model of Rejection‐related emotions for tDCS group and rejection appraisal (i.e. perceived percentage of ball tosses received). Table S5. Effect of tDCS stimulation, Cyberball conditions and their interactions on rejection‐related emotion controlled for age, occupational status and presence of psychiatric comorbidities. Table S6. Effect of tDCS stimulation, Cyberball conditions and their interactions on rejection‐related emotion controlled for different pharmacological categories. Table S7. Effect of tDCS stimulation, Cyberball conditions and their interactions on subscale of Rejected Emotion Scale. [file PCN-79-239-s001.docx]

**Reduction of rejection-related emotions by transcranial Direct Current Stimulation over right ventrolateral prefrontal cortex in borderline personality disorder:**

**A double-blind randomized pilot study**

**Short running title: tDCS reduces rejection feelings in BPD**

Alessandro Lisco MD, Alessia Gallucci PhD, Chiara Fabietti MD, Annalisa Fornaroli MD, Carlo Marchesi MD, Emanuele Preti PhD, Paolo Riva PhD, Chiara De Panfilis MD, Leonor Josefina Romero Lauro PhD

**Supplementary Material**

Due to journal word limitations, additional detailed descriptions that were omitted in the main text are provided below.

**Sample selection**

The sample size (N = 40) used in our study was based on a-priori power analysis performed with G-Power^1^ considering a Mixed Anova repeated measure, with tDCS (2 levels: real vs Sham) as between factor and *Cyberball* Condition (3 levels: inclusion, overinclusion and exclusion) as within subjects factor, with a mean effect size of 0.28 based on the effect sizes of our previous works^2-4^, α = .05, power =.95). Moreover, the same sample size was adopted in our previous works, thus allowing us to compare the present results on a clinical sample with those achieved on healthy participants.

We report below the effect sizes based on three of our previous tDCS research that also used 20 participants per group:

• Riva, P., Romero Lauro, L. J. R., DeWall, C. N., & Bushman, B. J. (2012). Buffer the pain away stimulating the right ventrolateral prefrontal cortex reduces pain following social exclusion. *Psychological Science, 23*, 1473-1475.

There were two key interactions in this study. The first, between tDCS and the manipulation of inclusionary status on pain unpleasantness, yielded a partial η2=.09 (Effect size f=0.31). The second, between tDCS and the manipulation of inclusionary status on hurt feelings, yielded a partial η2=.10 (Effect size f=0.33).

• Riva, P., Romero Lauro, L. J. R., DeWall, C. N., Chester, D. S., & Bushman, B. J. (2014). Reducing aggressive responses to social exclusion using transcranial direct current stimulation (tDCS). *Social Cognitive and Affective Neuroscience, 10*, 352-356.

In this study, the key interaction between tDCS and the manipulation of inclusionary status on behavioral aggression yielded a partial η2=.065 (Effect size f=0.26).

• Riva, P., Romero Lauro, L. J., Vergallito, A., DeWall, C. N., & Bushman, B. J. (2015). Electrified emotions: Modulatory effects of transcranial direct stimulation on negative emotional reactions to social exclusion. *Social Neuroscience, 10*, 46-54.

There were three key interactions in this study. The first, between tDCS and the manipulation of inclusionary status on social pain, yielded a partial η2=.07 (Effect size f=0.27). The second, between tDCS and the manipulation of inclusionary status on hurt feelings, yielded a partial η2=.05 (Effect size f=0.23). The third interaction, between tDCS and the manipulation of inclusionary status on negative emotions, yielded a partial η2=.06 (Effect size f=0.25).

Interrater reliability for a diagnosis of Borderline Personality Disorder (BPD), the main inclusion criteria of this study, was good (mean intraclass correlation coefficient for BPD criterion count: .94; Cohen’s for a BPD diagnosis: 1)

**Baseline measures**

Before the experiment, the included participants completed the following self-report questionnaires assessing baseline characteristics of the sample (see Table S1):

The *Inventory of Personality Organization* (IPO; Lenzenweger et al., 2001; Prunas et al., 2012), a 57-item questionnaire (1 to 5 Likert scale), that measures the level of identity integration, quality of object relations, and primitive defenses.

The *Effortful Control Scale* (ECS) of the *Adult Temperament* (Evans & Rothbart, 2007), a 19-item questionnaire (1 to 7 Likert scale), that measures effortful control.

The *Difficulties in Emotion Regulation Scale* (DERS; Gratz & Roemer, 2004), a 36-item questionnaire (1 to 5 Likert scale), that assesses multiple aspects of emotional dysregulation;

The *Acceptance and Action Questionnaire* (AAQ-II; Bond, Hayes, Baer et al., 2011), a 7-item questionnaire (1 to 7 Likert scale), that measures the willingness to experience unwanted private events in the pursuit of one’s values and goals.

The *Justice Sensitivity Questionnaire* (JSQ; Schmitt et al., 2005; 2010), a 40-item questionnaire (1 to 5 Likert scale), that assesses victim sensitivity, beneficiary sensitivity, perpetrator sensitivity, and observer sensitivity.

The *Adult Rejection Sensitivity Questionnaire* (A-RSQ; Berenson et al., 2009), a 27-item questionnaire (1 to 6 Likert scale), that evaluates anxious and angry expectation about being accepted or rejected by significant others in social interactions.

The scores of the questionnaires are reported in Table S1 for both the Real and the Sham groups, with no between-groups differences on any measure.

**Additional analyses**

**Effect of tDCS on rejection appraisal**

To clarify whether tDCS condition had any effect on rejection appraisal, patients’ perception of ball tosses percentages during the experiment were entered as a dependent variable in a linear regression analysis, and the tDCS condition was entered as the independent variable (perceived ball tosses percentages ~ tDCS Group, table S2). The model was not significant [R^2^=0; F=.036, *p*=.85]. To further clarify whether any effect of tDCS upon (cognitive) rejection appraisal could emerge when controlling for the different phases of the experiment, we run a second regression analysis, entering perceived percentages of ball tosses as the dependent variable, and tDCS condition and actual ball tosses percentage received as the independent variables (perceived ball tosses percentages ~ tDCS Group*actual ball tosses percentage received; see Table S3 and Figure S1). The model was significant, with perceived ball tosses percentages being positively predicted only by actual ball tosses percentages received [R^2^=.538 F=44.94, p<.001]. Crucially, no effect of tDCS or of its interaction with actual ball tosses percentage received was found: therefore, tDCS condition had no effect on the participants’ (cognitive) rejection appraisal, as already shown by ANOVA (see results, Manipulation check).

**Effect of tDCS and rejection appraisal on Rejection-related emotion**

To clarify whether the effect of tDCS on rejection-related emotions was influenced by variations in the cognitive appraisal of rejection in this sample of BPD patients, we firstly entered RES mean values during the experiment as the dependent variable in a multiple regression analysis, and rejection appraisal (i.e., perceived percentage of ball tosses received), tDCS condition and their interactions as independent variables (RES ~ perceived ball tossess percentages*tDCS Group; see Table S4 and Figure S2). According to our model, the levels of rejection-related emotions were negatively predicted by the perceived percentage of ball tosses received, and by the tDCS condition [R^2^=.493; F=37.67, p<.001]. Importantly, the interaction between the perceived percentage of ball tosses received and the tDCS condition was not significant. Thus, this pattern of results confirms that, overall, greater rejection appraisal predicted greater levels of rejection-related emotions, as already indicated by the ANOVA; furthermore, the reduction of rejection-related emotions by tDCS is not explained by varying levels of (cognitive) rejection appraisal during the experiment.

**Effects of tDCS on rejection-related emotions controlling for age, occupational status and psychiatric comorbidities**

A series of 2x3 mixed model analysis of co-variance (ANCOVA) was performed, with the randomized stimulation condition (2 groups: Real vs. Sham tDCS) as the between-subjects factor, the *Cyberball* conditions (3 conditions: inclusion, exclusion, and over-inclusion) as the within-subjects factor and, respectively, age (in years), occupational status (employed vs unemployed) and presence of a psychiatric comorbidity as a covariate. The models confirmed the results of the main analysis (see Table S5).

In particular, the ANCOVA confirmed a significant main effect of the *Cyberball* condition on RES total score, with participants reporting progressively lower levels of rejection-related emotions from the exclusion condition to the inclusion condition to the overinclusion condition.

Secondly, the analysis confirmed a significant main effect of tDCS condition: patients in the Real group reported lower levels of rejection-related emotions than those in the Sham group. Conversely, there was no significant main effect for the covariates considered.

Thirdly, a significant *Cyberball*XtDCS interaction also emerged. In particular, patients in the Real group, as compared to patients in the Sham group, reported fewer rejection-related emotions in both the exclusion (respectively: *p*=.025 with age as a covariate; *p*=.022 with occupational status; *p*=.029 with presence of comorbidities) and the inclusion (respectively: *p*=.005 with age as a covariate; *p*=.005 with occupational status; *p*=.004 with presence of comorbidities) conditions, while the two groups did not differ in the over-inclusion condition (respectively: *p*=.824 with age as a covariate; *p*=.740 with occupational status; *p*=.891 with presence of comorbidities) (Effect of Group within the GroupXCondition interaction). The effect of Condition within the GroupXCondition interaction further clarified that, while participants in the Sham group experienced fewer rejection-related emotions in the over-inclusion condition, as compared to inclusion (all ps<.001), participants in the Real group reported similar levels of rejection-related emotions in the fair inclusion and over-inclusion conditions (respectively: *p*=.525 with age as a covariate; *p*=.508 with occupational status; *p*=.648 with presence of comorbidities), suggesting that, in BPD patients, the Real tDCS stimulation was effective in decreasing the negative emotions elicited by fair inclusion to levels comparable to those of the over-inclusion condition regardless of psychiatric comorbidities and demographic variables.

**Effects of tDCS on rejection-related emotions controlling for different pharmacological classes**

A series of 2X3 mixed model analysis of co-variance (ANCOVA) was performed, with the randomized stimulation condition (2 groups: Real vs. Sham tDCS) as the between-subjects factor, the *Cyberball* conditions (3 conditions: inclusion, exclusion, and over-inclusion) as the within-subjects factor and, respectively, anxiolytics, antidepressant, mood stabilizers and antipsychotics drug use (yes vs no) as a covariate (see Table S6).

There was no main effect of any pharmacological category on RES scores.

The ANCOVA further confirmed a significant main effect of the *Cyberball* condition on RES total score even when controlling for the different medication types, with participants reporting progressively lower levels of rejection-related emotions from the exclusion condition to the inclusion condition to the overinclusion condition.

Similarly, the analysis confirmed a significant main effect of tDCS condition independent of the medication status, with patients in the Real group reporting lower levels of rejection-related emotions than those in the Sham group.

Thirdly, a significant *Cyberball*XtDCS interaction also emerged for the Analyses where antidepressant and anxiolytic use was entered as a covariate. In particular, patients in the Real group, as compared to patients in the Sham group, reported fewer rejection-related emotions in both the exclusion (both *ps*=.02) and the inclusion (both *ps*=.01) conditions, while the two groups did not differ in the over-inclusion condition (respectively: *p*=.72 for anxiolytics and *p*=.75 for antidepressant use) (Effect of Group within the GroupXCondition interaction). The effect of Condition within the GroupXCondition interaction further clarified that, for both antidepressants and anxiolytics use, while participants in the Sham group experienced fewer rejection-related emotions in the over-inclusion condition, as compared to inclusion (both ps<.001), participants in the Real group reported similar levels of rejection-related emotions in the fair inclusion and over-inclusion conditions (respectively: *p*=.423 for anxiolytics and *p*=.525 for antidepressant use), suggesting that, in BPD patients, the Real tDCS stimulation was effective in decreasing the negative emotions elicited by fair inclusion to levels comparable to those of the over-inclusion condition controlling for use of anxyolitics and antidepressant use. Conversely, the *Cyberball*XtDCS interaction was not significant when controlling for mood stabilizers and antipsychotic use (both *p=.*06).

Notably, we recommend caution when interpreting these results since the size each specific medication subgroup was limited.

**Effect of tDCS on RES individual subscales**

The ANOVA showed a significant main effect of the *Cyberball* condition for all RES subscales (see table S7): in particular, participants reported higher levels of anger, hurt feelings, sadness, and anxiety in the exclusion condition compared to both inclusion and overinclusion conditions, with no differences between inclusion and overinclusion. Moreover, participants felt progressively less rejected and happier from the exclusion condition to the inclusion condition to the overinclusion condition.

Secondly, the analysis showed a significant main effect of tDCS condition for rejection feelings, anger, sadness and anxiety, with patients in the Real group reporting lower levels of the aforementioned emotions than those in the Sham group (Table S7).

Thirdly, significant *Cyberball*XtDCS interactions for rejection feelings, anger and sadness also emerged.

For rejection feelings, the Real group felt less rejected in the inclusion condition (*p=.*003), compared to the sham group. The two groups did not differ in the exclusion and over-inclusion conditions (all *ps>*.05) (Effect of Group within the GroupXCondition interaction)

As for sadness, patients in the Real group, as compared to patients in the Sham group, reported lesser feelings of sadness in the exclusion condition (*p=*.012) (Effect of Group within the GroupXCondition interaction). The effect of Condition within the GroupXCondition interaction further clarified that, while participants in the Sham group experienced higher sadness in the exclusion condition, as compared to inclusion and overinclusion conditions (both *ps*<.001), participants in the Real group reported similar levels of sadness in all game conditions (all *ps>*.05).

Finally, the Real group felt less angry in both the exclusion and the inclusion conditions (*p*=.01; *p*=.01), compared to the Sham group. The two groups did not differ in the over-inclusion condition (all *ps>*.05) (Effect of Group within the GroupXCondition interaction). Moreover, participants in the Real group reported higher levels of angry feelings in the exclusion condition as compared to the inclusion condition (*p*=.01), with no differences between the overinclusion condition and both the inclusion (*p*=1) and the exclusion (*p*=.07) conditions. Conversely, participants in the Sham group reported significant higher levels of anger in the exclusion condition compared to both inclusion and overinclusion condition (both *ps<*.001), with no differences between inclusion and overinclusion (*p*=.14) (Effect of Condition within the GroupXCondition interaction).

**References**

1. Faul F, Erdfelder E, Lang AG, Buchner A. G*Power 3: a flexible statistical power analysis program for the social, behavioral, and biomedical sciences. *Behav. Res. Methods*. 2007; **39**: 175-91.
2. Riva P, Romero Lauro LJ, Dewall CN, Bushman BJ. Buffer the pain away: stimulating the right ventrolateral prefrontal cortex reduces pain following social exclusion. *Psychol. Sci.* 2012; **23**: 1473-5.
3. Riva P, Romero Lauro LJ, DeWall CN, Chester DS, Bushman BJ. Reducing aggressive responses to social exclusion using transcranial direct current stimulation. *Soc. Cogn. Affect. Neurosci.* 2015; **10**: 352-6.
4. Riva P, Romero Lauro LJ, Vergallito A, DeWall CN, Bushman BJ. Electrified emotions: Modulatory effects of transcranial direct stimulation on negative emotional reactions to social exclusion. *Soc. Neurosci.* 2015; **10**: 46-54.

**Figures**

**Fig S1. Interaction between rejection appraisal (perceived ball tosses received) and actual ball tosses received for tDCS groups.**


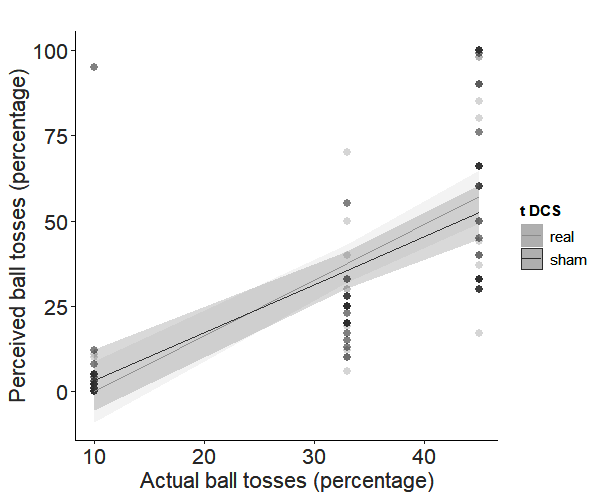


**Fig S2. Interaction between rejection appraisal (perceived ball tosses received) and Rejection-related emotions for tDCS groups.**

**
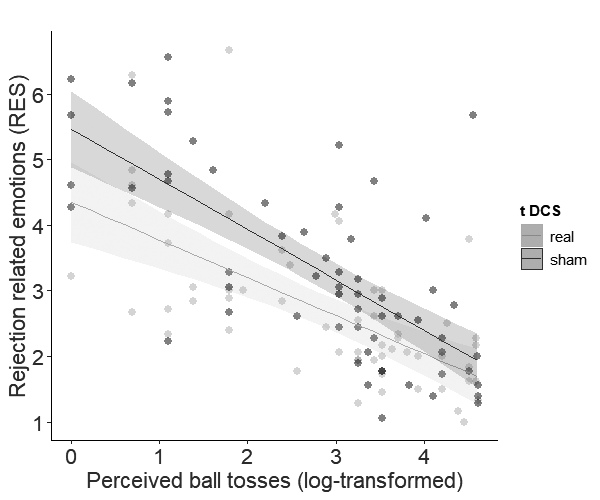
**

**Tables**

**Table S1. Sample baseline measures and comparison**

|  | | **Real group (n = 20)** | **Sham group(n = 20)** | ***T*** | ***p*** |
| --- | --- | --- | --- | --- | --- |
| IPO | |  |  |  |  |
|  | Identity | 3.04 (±,96) | 3.42 (±.78) | -1.364 | .18 |
|  | Primitive defenses | 3.03 (±,87) | 3.43 (±.79) | -1,539 | .13 |
|  | Reality | 2.38 (±,87) | 2.56 (±.66) | -.76 | .45 |
|  |  |  |  |  |  |
| ECS (mean) | | 3.97 (±1.16) | 3.53 (±.78) | 1,42 | .164 |
| DERS (total) | | 56.95 (±14.75) | 62.3 (±11.14) | -1,295 | .20 |
| AAQ-II (total) | | 34.25 (±11.4) | 36.3 (±11.14) | -,575 | .57 |
|  | |  |  |  |  |
| JSQ | |  |  |  |  |
|  | Victim sensitivity | 3.23 (±1.24) | 3.71 (±1.34) | 1,174 | .25 |
|  | Beneficiary sensitivity | 3.11 (±.99) | 2.74 (±1.2) | 1,043 | .30 |
|  | Perpetrator sensitivity | 3.92 (±1) | 3.31 (±1.3) | 1,676 | .10 |
|  | Observer sensitivity | 3.23 (±1.15) | 3.22 (±1.43) | ,183 | .86 |
|  |  |  |  |  |  |
| A-RSQ | |  |  |  |  |
|  | Anxious expectation of being rejected | 11,47 (±5.27) | 10,04 (±4.08) | ,958 | .34 |
|  | Angry expectation of being rejected | 7.28 (±3.94) | 8.01 (±2.87) | -,662 | .51 |

Notes. p-values of significant differences are in bold

**Table S2. Regression Model of perceived percentage of ball tosses received for tDCS Group**

|  | **B** | **SE B** | **β** | ***p*** |
| --- | --- | --- | --- | --- |
| Constant | 31,52 | 3,93 |  |  |
| tDCS group (Sham vs Real) | -1,05 | 5,55 | -.017 | .85 |

Note: (p<.001)

**Table S3. Regression Model of perceived percentage of ball tosses received for tDCS Group and actual ball tossess received**

|  | **B** | **SE B** | **β** | ***p*** |
| --- | --- | --- | --- | --- |
| Constant | -16.38 | 6,07 |  |  |
| tDCS Group (Sham vs Real) | 5.70 | 8,58 | .09 | .508 |
| Actual percentage of ball tosses received | 1.63 | 0,18 | .79 | **<.001** |
| tDCS group x Actual percentage of ball tosses received | -0.23 | 0,26 | -.14 | .382 |

Note: (p<.001)

**Table S4. Regression Model of Rejection-related emotions for tDCS Group and rejection appraisal (i.e., perceived percentage of ball tosses received)**

|  | **B** | **SE B** | **β** | ***p*** |
| --- | --- | --- | --- | --- |
| Constant | 4,35 | 0,31 |  |  |
| tDCS Group (Sham vs Real) | 1.11 | 0,42 | .04 | **.010** |
| Perceived percentage of ball tosses received (natural logaritm) | -0.58 | 0,1 | -.56 | **<.001** |
| tDCS group x Perceived percentage of ball tosses received | -0.19 | 0,14 | -.24 | .169 |

Note: (p < .001)

**Table S5.** **Effect of tDCS stimulation, Cyberball conditions and their interactions on Rejection-related emotion controlled for age, occupational status and presence of psychiatric comorbidities**

|  |  |  |  |  |  |
| --- | --- | --- | --- | --- | --- |
|  | **Cyberball** | **tDCS** | **Covariate** | **Cyberball X tDCS** | **Cyberball X Covariate** |
| **RES by age** | F(1.908, 70.58)=8.524  η^2^*_p_*=.19  ***p*=.001^a^** | F(1, 37)=7.117  η^2^*_p_*=.16  ***p*=.01^b^** | F(1, 37)=2.64  η^2^*_p_*=.07  *p*=.11 | F(1.87, 71.055)=3.235  η^2^*_p_*=.08  ***p*=.05^cd^** | F(1.87, 71.055)=.046  η^2^*_p_*=.0  *p*=.95 |
| **RES by occupational status (employed/unemployed)** | F(1.907, 70.555)=55.004  η^2^*_p_*=.60  ***p*<.001^a^** | F(1, 37)=7.258  η^2^*_p_*=.16  ***p*=.01^b^** | F(1, 37)=.056  η^2^*_p_*=.0  *p*=.81 | F(1.87, 71.055)=3.206  η^2^*_p_*=.08  ***p*=.05^cd^** | F(1.87, 71.055)=.058  η^2^*_p_*=.0  *p*=.93 |
| **RES by presence of comorbidities (yes/no)** | F(1.829, 67.688)=38.89  η^2^*_p_*=.512  ***p*<.001^a^** | F(1, 37)=6.716  η^2^*_p_*=.15  ***p*=.01^b^** | F(1, 37)=.285  η^2^*_p_*=.01  *p*=.60 | F(1.829, 67.688)=3.54  η^2^*_p_*=.09  ***p*=.04^cd^** | F(1.829, 67.688)=1.467  η^2^*_p_*=.04  *p*=.24 |

Notes. p-values of significant effects and interactions are in bold.

Significant post-hoc (all p_s_<.05) expressed with the following apex indices:

a= exclusion>inclusion>over-inclusion

b= real<sham

c= real: exclusion>inclusion=over-inclusion; sham: exclusion>inclusion>over-inclusion

d= inclusion and exclusion: real<sham; over-inclusion: real=sham

**Table S6.** **Effect of tDCS stimulation, Cyberball conditions and their interactions on Rejection-related emotion controlled for different pharmacological categories**

|  |  |  |  |  |  |
| --- | --- | --- | --- | --- | --- |
|  | **Cyberball** | **tDCS** | **Covariate** | **Cyberball X tDCS** | **Cyberball X Covariate** |
| **RES by Anxiolytics (yes/no)** | F(1.821, 67.388)=33.9  η^2^*_p_*=.48  ***p*<.001^a^** | F(1, 37)=7.186  η^2^*_p_*=.16  ***p*=.01^b^** | F(1, 37)=.033  η^2^*_p_*=.0  *p*=.86 | F(1.821, 67.388)=3.309  η^2^*_p_*=.08  ***p*=.05^cd^** | F(1.821, 67.388)=1.73  η^2^*_p_*=.04  *p*=.188 |
| **RES by Antidepressants (yes/no)** | F(1.903, 70.396)=25.997  η^2^*_p_*=.41  ***p*<.001^a^** | F(1, 37)=7.265  η^2^*_p_*=.16  ***p*=.01^b^** | F(1, 37)=.015  η^2^*_p_*=.0  *p*=.90 | F(1.903, 70.396)=3.247  η^2^*_p_*=.08  ***p*=.05^cd^** | F(1.903, 70.396)=.134  η^2^*_p_*=.0  *p*=.86 |
| **RES by Mood Stabilizers (yes/no)** | F(1.907, 70.563)=29.514  η^2^*_p_*=.44  ***p*<.001^a^** | F(1, 37)=9.648  η^2^*_p_*=.21  ***p*=.01^b^** | F(1, 37)=2.327  η^2^*_p_*=.06  *p*=.14 | F(1.907, 70.563)=2.876  η^2^*_p_*=.07  *p=.*06 | F(1.907, 70.563)=.034  η^2^*_p_*=.0  *p=.*96 |
| **RES by Antipsychotics (yes/no)** | F(1.914, 70.83)=36.422  η^2^*_p_*=.496  ***p*<.001^a^** | F(1, 37)=7.739  η^2^*_p_*=.17  ***p*=.01^b^** | F(1, 37)=.633  η^2^*_p_*=.02  *p*=.43 | F(1.914, 70.83)=2.955  η^2^*_p_*=.07  *p*=.06 | F(1.914, 70.83)=.514  η^2^*_p_*=.01  *p*=.59 |

Notes. p-values of significant effects and interactions are in bold.

Significant post-hoc (all p_s_<.05) expressed with the following apex indices:

a= exclusion>inclusion>over-inclusion

b= real<sham

c= real: exclusion>inclusion=over-inclusion; sham: exclusion>inclusion>over-inclusion

d= inclusion and exclusion: real<sham; over-inclusion: real=sham

**Table S7.** **Effect of tDCS stimulation, Cyberball conditions and their interactions on subscale of Rejected Emotion Scale**

|  | **Real tDCS (n=20)** | | | **Sham tDCS (n=20)** | | |  |  |  |
| --- | --- | --- | --- | --- | --- | --- | --- | --- | --- |
|  | **Inclusion** | **Exclusion** | **Over-**  **inclusion** | **Inclusion** | **Exclusion** | **Over-**  **inclusion** | **Cyberball** | **tDCS** | **Cyberball X tDCS** |
| **Rejection** | 2.78 (1.41) | 6.13 (1.11) | 1.55 (.65) | 4.33 (1.63) | 6.33 (1.35) | 1.65 (.79) | F(2, 76)=163.922  η^2^*_p_*=.81  ***p*<.001^a^** | F(1, 38)=6.61  η^2^*_p_*=.15  ***p*=.01^d^** | F(2, 76)=4.968  η^2^*_p_*=.12  ***p*=.01^e^** |
| **Anger** | 1.35 (.67) | 2.55 (1.82) | 1.55 (1.3) | 2.13 (1.16) | 4.22 (2.08) | 1.52 (.83) | F(2, 76)=28.87  η^2^*_p_*=.43  ***p*<.001^b^** | F(1, 38)=6.35  η^2^*_p_*=.15  ***p*=.02^d^** | F(2, 76)=4.97  η^2^*_p_*=.12  ***p*=.01^efg^** |
| **Sadness** | 1.60 (1.08) | 2.13 (1.44) | 1.35 (.52) | 2.15 (1.27) | 3.52 (1.86) | 1.55 (1.07) | F(1.868; 70.971)=20.321  η^2^*_p_*=.35  ***p*<.001^b^** | F(1, 38)=5.188  η^2^*_p_*=.12  ***p*=.03^d^** | F(1.868; 70.971)=3.788  η^2^*_p_*=.09  ***p*=.03^fh^** |
| **Anxiety** | 1.85 (1.05) | 3.02 (1.87) | 1.97 (1.55) | 2.85 (1.42) | 3.92 (1.74) | 2.45 (1.62) | F(2, 76)=14.608  η^2^*_p_*=.28  ***p*<.001^b^** | F(1, 38)=3.994  η^2^*_p_*=.09  ***p*=.05^d^** | F(2, 76)=.58  η^2^*_p_*=.01  *p*=.56 |
| **Hurt Feelings** | 1.38 (.78) | 2.15 (1.74) | 1.32 (.63) | 1.73 (1.11) | 3.23 (2.09) | 1.25 (.72) | F(1.44, 54.724)=19.224  η^2^*_p_*=.34  ***p*<.001^b^** | F(1, 38)=2.245  η^2^*_p_*=.06  *p*=.14 | F(1.44, 54.724)=2.924  η^2^*_p_*=.07  *p*=.08 |
| **Happiness** | 2.82 (1.57) | 1.8 (1.1) | 3.5 (1.68) | 2.32 (1.14) | 1.3 (.52) | 3.7 (1.85) | F(1.87, 71.055)=31.911  η^2^*_p_*=.46  ***p*<.001^c^** | F(1, 38)=.687  η^2^*_p_*=.02  *p*=.41 | F(1.87, 71.055)=1.24  η^2^*_p_*=.03  *p*=.29 |

Notes.

p-values of significant effects and interactions are in bold. Significant post-hoc (all p_s_<.050) expressed with the following apex indices:

a= exclusion>inclusion>overinclusion

b= exclusion>inclusion=overinclusion

c= exclusion<inclusion<overinclusion

d= real < sham

e= inclusion: real<sham

f= exclusion: real<sham

g= real: exclusion=overinclusion, inclusion=overinclusion, exclusion>inclusion; sham: exclusion>inclusion=overinclusion

h= real: exclusion=inclusion=overinclusion; sham: exclusion>inclusion=overinclusion
